# Meningoencephalitis in Flea-Borne Typhus: A Report of Two Cases and a Review of CNS Complications

**DOI:** 10.3390/pathogens15060590

**Published:** 2026-05-30

**Authors:** Camille E. Spears, Divya Chandramohan, Melinda B. Tanabe, Nicholas C. Anstead, Emily L. M. Turkily, Lucas S. Blanton, Thelma Akien, Christopher L. Dayton, James C. Saca, Gregory M. Anstead

**Affiliations:** 1 Division of Infectious Diseases, Department of Medicine, University of Texas Health San Antonio, 7703 Floyd Curl Drive, San Antonio, TX 78229, USA; spears@uthscsa.edu (C.E.S.); chandramohan@uthscsa.edu (D.C.); 2 Division of Infectious Diseases, Department of Medicine, University of Texas Medical Branch, Galveston, TX 77555, USA; mbtanabe@utmb.edu (M.B.T.); lsblanto@utmb.edu (L.S.B.); 3 Department of Psychological Sciences, Texas Tech University, Box 42051, Lubbock, TX 79409, USA; nianstea@ttu.edu (N.C.A.); eturkily@ttu.edu (E.L.M.T.); 4 Cleveland Clinic Foundation, 10300 Carnegie Ave, Cleveland, OH 44106, USA; dangant@ccf.org; 5 Department of Emergency Medicine, University of Texas San Antonio, 7703 Floyd Curl Dr., San Antonio, TX 78229, USA; dayton@uthscsa.edu; 6 Division of Critical Care and Pulmonology, Department of Medicine, University of Texas San Antonio, 7703 Floyd Curl Dr., San Antonio, TX 78229, USA; 7 Division of Hospital Medicine, University of Texas San Antonio, 7703 Floyd Curl Drive, San Antonio, TX 78229, USA; sacaj@uthscsa.edu; 8 Division of Infectious Diseases, Medical Service, South Texas Veterans Health Care System, 7400 Merton Minter Blvd, San Antonio, TX 78229, USA

**Keywords:** flea-borne typhus, *Rickettsia typhi*, meningitis, encephalitis, meningoencephalitis, seizures, cranial nerve palsies, plasmacytoid lymphocyte

## Abstract

Typhus (FBT), caused by *Rickettsia typhi*, rarely causes neurological disease. Herein, we describe neurological involvement in two cases of FBT. In the first case, an adult presented with persistent fever which deteriorated into status epilepticus. He was successfully treated with doxycycline and made a complete recovery. In the second case, a patient suffered an ischemic stroke and had a protracted clinical course but ultimately made a near complete recovery. In addition to these cases, we conducted a comprehensive narrative review of 43 cases of neurologic involvement in FBT reported from 1989 to 2025. Cases were excluded if there were pathologic discrepancies with typical cases of FBT. Presentations ranged from cranial nerve palsies to meningitis and fulminant encephalitis. This review highlights the spectrum of CNS complications associated with FBT and underscores the importance of early recognition and treatment with doxycycline to improve outcomes. Given the potential severity of neurologic involvement, clinicians in endemic areas should maintain a high index of suspicion for FBT in patients presenting with a febrile illness and neurologic symptoms.

## 1. Introduction

Flea-borne typhus (FBT) is an infection caused by the bacterium *Rickettsia typhi*. It is typically an acute undifferentiated febrile illness, but about one-quarter of patients suffer from respiratory, neurologic, hematologic, renal, hepatic, cardiac, ocular, or other complications [1]. About one-third of hospitalized adult patients stricken with FBT require intensive care [2]. The infection is most commonly transmitted to humans by inoculation of a bite site, a skin abrasion, or mucous membranes with feces from fleas infected with these rickettsiae [3,4].

FBT is the most prevalent and widely distributed rickettsiosis; it occurs on every continent except Antarctica [3]. In the United States as a whole, FBT is now uncommon, but foci still exist in Texas, Hawaii, and California [5]. In the last decade, the incidence of FBT has increased in both Texas and California [6,7]. Outside of the United States, FBT is re-emerging in multiple locations due to changing environmental conditions promoting increased levels of transmission [8,9]. As the number of cases of FBT increases, it is important for clinicians to recognize its possible neurologic manifestations and initiate appropriate and timely treatment to decrease morbidity and potential long-term neurologic sequelae.

The aim of the current study is to report two cases of FBT that presented as encephalitis. In addition, we reviewed and summarized 43 other cases of FBT with neurological symptoms described in the medical literature from 1989 to 2025. To determine the approximate incidence of FBT CNS disease, we examined 31 case series from 1945 to 2025, comprising 2508 patients.

## 2. Methods of the Narrative Review

To find cases of FBT involving the central nervous system and case series, PubMed was searched for articles published from 1 January 1945 to 1 July 2025 using the terms (“meningitis” or “encephalitis” or “meningoencephalitis”) and (“flea-borne typhus” or “murine typhus” or “endemic typhus”). Additional references were found by Google Scholar searches using identical search terms and bibliographic branching. References published in English, French, and Spanish were examined. The included identified cases were considered probable or confirmed by the original investigators. Cases were excluded if they had pathologic discrepancies with typical cases of FBT (as described in Section 4.2). We excluded five cases of cerebral vasculitis reported by Wenzel and coworkers due to uncertainty regarding the serologic diagnoses [10]. Cases specifically identified as due to *R. felis* were excluded (these will be addressed in a future publication [Alison Wiseman, personal communication]). Cases of retinitis or hearing loss without other CNS manifestations were also excluded. For the case review, we have focused on neurologic presentations and complications.

Encephalitis was defined by lethargy, stupor, coma, seizures, neuropsychiatric changes, cranial nerve dysfunctions, other focal neurological deficits, movement disorders, ataxia, and/or hemiparesis [11]. Meningitis refers to headaches with abnormal cerebrospinal fluid (CSF) findings. Meningoencephalitis is defined as the signs and symptoms of encephalitis and abnormal CSF findings. Some cases of encephalitis may be meningoencephalitis, but if a lumbar puncture was not performed, they are classified as encephalitis. Pleocytosis and elevated CSF protein are defined as a CSF WBC count > 5/μL and >45 mg/dL, respectively. Hypoglycorrhachia is defined as a CSF glucose level of ≤45 mg/dL or a CSF to serum glucose ratio ≤ 0.5. However, the CSF/serum glucose ratio may be unreliable if the two determinations were not performed within 60 min of each other [12]. Statistical analyses were conducted in R, version 4.5.2 (R Core Team, Vienna, Austria, 2025; www.Rproject.org). Statistical significance was evaluated at *p* < 0.05.

## 3. Case Presentations

### 3.1. Case Report #1

A 40-year-old male, a resident of San Antonio, Texas, with well-controlled epilepsy, with the last seizure occurring over a decade ago, continued on divalproex 250 mg oral extended-release tablet daily, presented to the hospital with a chief complaint of fevers, after having spent eight days in Panama visiting the Panama Canal, and some time in urban areas in Colombia. He owned three dogs and a cat without flea prevention. Six weeks after his trip, he developed fevers up to 40 °C, chills, night sweats, productive cough, headaches, and loose stools for one and a half weeks. He identified as a gay male in a monogamous relationship for about ten years, with no prior history of sexually transmitted infections. Ten days prior to hospital presentation, he tested negative for coronavirus disease-19 (COVID-19) and influenza A/B with a nasopharyngeal screen. He was empirically treated with a course of azithromycin and prednisone and was sent to the hospital for further evaluation due to continued high fevers. His physical exam was unremarkable on arrival. He had two witnessed generalized tonic–clonic seizures followed by a prolonged post-ictal altered state, which raised concerns for status epilepticus. This prompted emergent intubation for airway protection. After administration of levetiracetam, valproic acid, and lacosamide, his seizures were controlled. Electroencephalography thereafter showed a continuous background of predominantly theta activity with intermixed delta activity that was poorly reactive, consistent with stupor. He had fevers up to 39.4 °C and was hemodynamically stable. Laboratory evaluation revealed a white blood cell (WBC) count of 18.1 K/mL (reference range (RR) 3.4–10.4 K/mL) with a neutrophil predominance of 85%, a platelet count of 73 K/mL (RR 140–377 K/mL), creatinine of 1.4 mg/dL (RR 0.6–1.3 mg/dL), alanine aminotransferase (ALT) 158 U/L (RR < 46 U/L), aspartate aminotransferase (AST) 162 U/L (RR < 35 U/L), alkaline phosphatase 200 U/L (RR 45–117 U/L), albumin 2.8 g/L (RR 3.6–5.1 g/dL), and total bilirubin 0.5 mg/dL (RR 0.2–1.2 mg/dL). He had a lactate dehydrogenase level of 321 U/L (RR 92–240 U/L) and did not develop hyponatremia. Additional testing included a C-reactive protein (CRP) of 98.7 mg/L (RR 0–10 mg/L) and positive parainfluenza virus on nasopharyngeal swab respiratory polymerase chain reaction (PCR) testing. His urinalysis was positive for 1+ protein on dipstick analysis, with no other abnormalities noted. The lactic acid level was 10.7 mmol/L (RR 0.5–2.0 mmol/L). A chest X-ray on the day of admission showed pulmonary edema, a right upper quadrant ultrasound was unremarkable, and a non-contrast head computed tomography (CT) was negative for abnormalities. He was started on acyclovir, vancomycin, and ceftriaxone as empiric therapies for meningitis and herpes encephalitis. Doxycycline was started on day 1 of the hospitalization for suspected FBT, and artemether–lumefantrine was initiated for empiric cerebral malaria treatment on day 2 of hospitalization.

**Figure 1:**
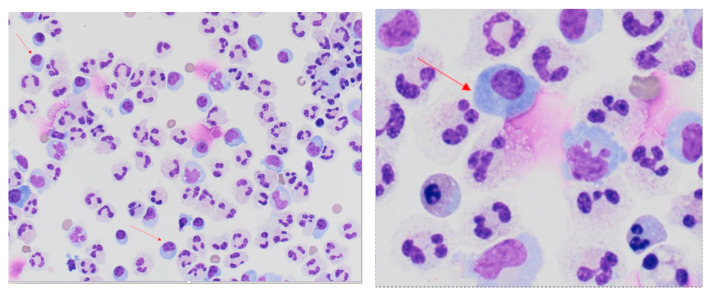
(**Left**): Plasma cells and plasmacytoid lymphocytes seen on CSF cytologic exam (hematoxylin and eosin (H&E), 200-× magnification). (**Right**): plasmacytoid lymphocyte (red arrow; H&E, 400-× magnification).

A CT chest obtained on the day of admission revealed bilateral posterior lower lobe atelectasis; a CT of the abdomen and pelvis revealed hepatosplenomegaly with diffuse periportal edema and perihepatic fluid, and enterocolitis in the distal ileum and ascending colon. The enterocolitis was explained by gastrointestinal PCR testing (Biofire FilmArray, BioFire Diagnostics; Salt Lake City, UT, USA), which was positive for astrovirus. Brain magnetic resonance imaging (MRI) performed with and without contrast showed no abnormality. A lumbar puncture revealed an elevated opening pressure, and cerebrospinal fluid (CSF) analysis demonstrated pleocytosis, an elevated protein level, and hypoglycorrhachia (Table 1). A CSF Gram stain and culture showed 4+ WBCs, but no growth. An extensive infectious evaluation for viral, bacterial, parasitic, and sexually transmitted pathogens was unrevealing, including repeated malaria smears, arboviral serologies, HIV testing, blood cultures, and CSF multiplex PCR testing (see Supplemental Materials). Plasma cells and plasmacytoid lymphocytes were seen on the CSF cytologic exam (Figure 1). The CSF cell counts and protein and glucose levels are shown in Table 1.

The fever curve improved on day four of the hospital course, and the leukocytosis, acute kidney injury, and encephalopathy resolved. Artemether–lumefantrine was discontinued after repeat malaria smears returned negative. A second LP performed on day 4 showed decreased opening pressure and improved cell counts (Table 1). He was extubated, following which he was alert and oriented, with persistent headaches, and without focal neurologic deficits. On hospital day six (about two weeks after symptom onset), *R. typhi* IgM and IgG resulted and were both 1:1024, consistent with a probable case of FBT meningoencephalitis per Centers for Diseases Control and Prevention guidelines; *R. rickettsii* IgG < 1:64, and IgM was 1:256 (negative < 1:64) (this is a serologic cross-reaction with *R. typhi*). Supporting the diagnosis was the observation that the patient displayed the constellation of laboratory findings typical of FBT, including elevated transaminase and LDH levels, a decreased albumin level, and thrombocytopenia, seen in 82.9%, 80.2%, 78.5%, and 49% of adult FBT patients, respectively [1]. All other antibiotics except doxycycline were discontinued, and he was discharged home with a ten-day course of doxycycline. Two weeks later, he was seen at a follow-up visit and was asymptomatic.

### 3.2. Case Report #2

A 68-year-old male with a past medical history of hypertension, heart failure with preserved ejection fraction, gout, hypertriglyceridemia, and type 2 diabetes presented to the emergency department with a two-day history of left anterior thigh pain, headaches, fatigue, myalgias, and high fevers (39.4 °C). His headache was frontal and throbbing, not associated with neck pain, photophobia, phonophobia, nausea, or vomiting. The patient was a resident of Galveston, Texas and had potential exposure to opossums and cats in his backyard. He had recently traveled to Las Vegas, Nevada and the California–Nevada border 3 weeks prior to symptom onset, where he went hiking in the desert and at Mount Charleston. On examination, he was diaphoretic and somnolent but arousable, with bilateral thigh tenderness and a diffuse macular rash on his abdomen. Initial blood work showed WBC 7.34 K/µL (RR 3.4–10.4 K/µL) with a left shift, a platelet count of 73 K/µL (RR 140–377 K/µL), sodium 132 mmol/L (RR 136–145 mmol/L), glucose 195 mg/dL (RR 70–110 mg/dL), AST 58 U/L (normal < 36 U/L), ALT 39 U/L (normal < 46 U/L), CK 694 U/L (RR 24–233 U/L), and CRP 7.4 mg/dL (RR < 0.8 mg/dL). Other lab testing revealed a negative urine toxicology screen for controlled substances, HIV and syphilis serologic tests, an influenza nasopharyngeal swab, blood cultures, and urine culture. The chest X-ray was unremarkable. An MRI of the left thigh revealed calcific trochanteric bursitis, tendinopathy, and muscle strain. On hospital day 5, the patient developed worsening somnolence and decreased responsiveness, new-onset pulmonary edema, atrial fibrillation, and hemodynamic instability requiring intubation, vasopressor support, and continuous renal replacement therapy.

On hospital day 5, doxycyline 100 mg every 12 h was started. A CT of the head revealed mild diffuse cerebral edema. A lumbar puncture showed 3 WBC/µL (93% lymphocytes), 1 red blood cell/µL, a glucose level of 131 mg/dL (serum glucose 217 mg/dL; ratio 0.60), and a protein level of 67 mg/dL. CSF Gram stain and bacterial, acid-fast bacilli, and fungal cultures resulted negative. A meningoencephalitis PCR panel (Biofire FilmArray, BioFire Diagnostics, Salt Lake City, UT, USA) was negative. An MRI of the brain found a small focus of restricted diffusion in the central pons. *Rickettsia typhi* serologic testing on hospital day 6 revealed IgM and IgG titers of >1:1024 and 1:128, respectively. Repeat *R. typhi* serologic testing on hospital day 14 demonstrated an unequivocal confirmatory IgG response (IgM and IgG titers of >1:1024 and 1:1024, respectively). The patient was treated with doxycycline until he had been afebrile for >48 h, receiving a total of 14 days. The patient remained obtunded for two weeks off sedating medications. A tracheostomy and gastric tube were placed, and he was weaned off mechanical ventilation. At the time of discharge to a rehabilitation facility, he was able to follow commands but was severely deconditioned. Two months later, the patient had made an almost complete recovery except for mild residual cognitive impairment.

## 4. Discussion

### 4.1. General Aspects of CNS Infection in Flea-Borne Typhus

This discussion has five goals. First, there is a general discussion of CNS involvement in FBT. The second goal is to place these two cases within the context of prior cases of FBT with neurologic involvement. To accomplish this task, we reviewed 43 previous cases of FBT with CNS manifestations. The third goal is to examine the case series reported in the literature to determine the frequency and nature of CNS involvement in FBT. The fourth goal is to describe the cytopathologic features of our Case Report #1. To our knowledge, the cytologic findings of the CSF in FBT have not been previously described. Finally, we discuss treatment strategies that may improve outcomes in FBT CNS disease, including a loading dose of doxycycline, using minocycline instead of doxycycline, and the concurrent use of corticosteroids.

Flea-borne typhus, also known as murine or endemic typhus, is caused by the obligately intracellular Gram-negative bacteria, *R. typhi*, which is transmitted by several species of fleas [13]. In Case Report #1, the source of the illness was likely cat flea exposure from dog and cat ownership. In the second case, the patient reported exposure to cats and opossums in his yard. The term typhus originates from the Greek word “typhos,” meaning heavy stupor, and is also related to “typhein,” which means smoke [14].

The word “Typhus” now refers to the illnesses caused by certain members of the genera *Rickettsia* and *Orientia*. These different infections do not cause neurological involvement to the same degree. In addition to louse-borne typhus, which played a role in the coining of the disease name, the greatest extent of neurological complications occurs in Rocky Mountain Spotted Fever (RMSF), followed by scrub typhus, due to *O. tsutsugamushi* [11,15]. Only eight autopsy cases of FBT have been reported in the medical literature [16,17,18,19,20,21]. Two of the autopsy cases did not display any neuropathological changes ascribed to FBT [16,19]. Gross abnormalities of the CNS described in three of these cases included mild to severe cerebral edema and congestion [16,18,22]. In one of the cases, mild cerebellar tonsillar herniation was observed [18]. Additional findings in the brain included multifocal petechiae in the white matter, ischemic necrosis of neurons within the cerebral and hippocampal watershed areas, mononuclear infiltration of the meninges, perivasculitis of the pituitary, and perivascular mononuclear infiltrates, the characteristic vascular insult associated with rickettsial infection [16,17,18,22].

In a previous study, a histopathologic assessment of brain sections in seven patients with typhus encephalitis, five from *R. prowazekii* infection and two due to FBT, revealed similarities between the two rickettsioses. These brain specimens showed nodular perivascular cell accumulations with CD4- and CD8-positive T cells, CD68-positive microglia, macrophages, and B-cells. Neutrophils were rarely found. These nodules were primarily T-cell-predominant and are called typhus (glial) nodules. All brain sections with *R. prowazekii* and *R. typhi* infection possessed these nodules, which were primarily observed in the pons and medulla. This perivascular inflammation is explained by the propensity of both *R. prowasekii* and *R. typhi* to invade the vascular endothelium [23]. However, in FBT, the accumulation of these glial cells and mononuclear cells around gray matter capillaries rarely progresses to thrombotic occlusion, microinfarctions, and microhemorrhages, explaining the rapid reversibility of neurological signs in most individuals with this infection with appropriate treatment [24]. Although neurologic complications are less common in FBT than in RMSF or epidemic typhus, the spectrum of reported CNS manifestations is broad and potentially severe, including meningitis, encephalitis, persistent cognitive impairment, optic neuritis, and cranial nerve palsies (Table 2). Cerebral infarction and/or hemorrhage have been reported in only a few cases [18,25,26]. These are extremely rare, perhaps given the lack of thrombotic occlusion around gray matter capillaries in the central nervous system (CNS), in contrast to its capacity for vascular occlusion in other organs [18,24]. The CNS manifestations of FBT present later than meningoencephalitis secondary to bacterial etiology, typically in the second week of illness, which could be a useful clinical indicator that rickettsial pathogenesis is at play [27,28].

In Case Report #1, parainfluenza virus and astrovirus were found by PCR testing on nasopharyngeal and stool specimens, respectively. However, it is doubtful that these two viruses were involved in the neuropathogenesis of this case. Parainfluenza has been rarely associated with neurologic syndromes, primarily in children [29]. Astrovirus encephalitis has been rarely reported in humans and it almost always occurs in persons with severe underlying immunosuppression [30]. This patient’s rapid neurologic improvement after treatment with doxycycline bolsters the contention that the encephalitis was due to FBT. In Case Report #2, vasculitis and autoimmune encephalitis (AE) would be included in the differential diagnosis of the patient’s clinical presentation. However, vasculitis and AE do not resolve spontaneously and the patient recovered without receiving any immunosuppressants [31].

**Table 2:** Case reports of flea-borne typhus with meningitis and meningoencephalitis ^a^.

Case # [Ref] Year	Age (yrs); Sex; Health Status; Final Diagnosis	Presentation and Initial Treatment	Studies	Course and Outcome
#Woo [32] 1988	58; M; health ns; Meningitis	Headaches for 3 d, vomiting, dull mentation, neck rigidity. Then fever. CSF suggested bacterial meningitis; given chloramp and PCN G. Four days later, improved. Chloramp stopped on day 10.	CSF: WBC 11,600/μL (90% pmns); prot 524; gluc 20.	Fever again; persistent pleocytosis. Given PCN G, cefuroxime, gentamicin. Then + serology (W-Ft); given tetracycline; after 4 days, fever resolved, full recovery. (Unlikely case of FBT)
#Jang [33] 2018	52; M; healthy; Brain abscess	Headache of 5 months duration; months prior he had an illness with a rash. Given ceftazidime, vancomycin, and ampicillin/sulbactam. After 19 days on antibiotics, headaches did not subside.	MRI: 2 cm abscess. CSF: WBC 440/μL (42% lymphs, 13% pmns); prot 107; gluc ratio 0.54	Repeat MRI: incomplete response. Antibiotics continued for total of 6 weeks. Positive IgM, negative IgG. No doxy given. Headaches resolved. (Unlikely case of FBT)
#1 [34] 1989	19; M; healthy; Encephalitis	Fever, headache, photophobia, weakness, confusion for 6 days. Received chloramp for 9 days, fever decreased. After discharge, weakness continued; had difficulty with simple tasks.	EEG: normal.	After 6 weeks, had slow mentation, emotional lability, and unsteady gait. Neurologic status normalized within a few weeks (RAID).
#2 [34] 1989	36; M; healthy; Encephalitis	Ten days of fever and headache. Fever resolved in 4 days without therapy, and discharged after 14 days. Ten days later, somnolent, poor concentration, and inappropriate behavior.	CT normal. CSF: WBC very few; normal prot and gluc; EEG: mild generalized slowing	Somnolence slow mentation, and aggression continued. Given tetracycline for 1 week, with recovery of mental function (RNR).
#3 [26] 1989	37; F; healthy; ME	Headache, photophobia, myalgias, malaise, respiratory symptoms, nuchal rigidity. Cefotaxime started and stopped after negative CSF cultures. Doxy was given for 8 days; started rifampin-isoniazid-ethambutol (RIE).	CSF: WBC 321/μL (97% lymphs); prot 53; gluc ratio 0.49	Patient had tremor, myoclonic jerks, confusion, hallucinations; started acyclovir; improved; stopped RIE when TB cultures returned negative (RNR).
#4 [11] 1991	60; F; health ns; ME, multiorgan failure	Ten days of fever, headache, myalgias. Treated w/TMP-SMX. Fever continued; developed diarrhea, hypotension, altered consciousness, and respiratory failure.	OP: 14; CSF: WBC 45 monos/μL, 9 pmns/μL; prot 185; gluc 18	Given ampicillin, amikacin; chloramp; improved consciousness; died of cardiac arrest (DEATH).
#5 [11] 1991	70; M; alcoholism; Meningitis	Presented with jaundice, acute kidney injury.	CSF: WBC 5 monos/μL, 10 pmns/μL; prot 104; gluc ratio 0.41	Given doxy (one dose); recovered (RNR).
#6 [11] 1991	36; M; health ns, ME	Rash, seizure.	CSF: WBC 27 monos/μL; prot 55; gluc ratio 0.5	Given doxy (one dose); recovered (RNR).
#7 [24] 1998.	22; F; health ns; ME, abducens n. palsy	Headache, malaise and low-grade fever for 2 weeks; then headache worsened, with diplopia and vomiting.	OP: 5; CSF: WBC 10/μL (90% monos); prot 75; gluc 64	Given doxy, recovered (RNR).
#8 [24] 1998.	18; F; health ns; Encephalitis with seizures, hemiparesis	Fever and abdominal pain for 14 days; then blurry vision, vomiting, nuchal rigidity, papilledema, hemiparesis. Given isoniazid and rifampin. Had seizures, hemiparesis, and drowsiness. Repeat LP showed persistently elevated OP with increasing WBC, prot.	CT head: normal. Initial LP: OP: 31; CSF: WBC 83–640/μL; (90–98% mononucl); prot 101; gluc 45	After 14 days, given doxy, then gradually improved; CSF normal within 21 days; discharged with mild hemiparesis (TCNR).
#9 [24] 1998.	25; F; health ns; Meningitis, optic neuritis	Headache and visual blurring for 21 days. On admission, florid bilateral papilledema.	OP: 22; CSF: WBC 16/μL (95% monos); prot 38; gluc 58	Doxy was started; headache resolved promptly, but the patient had residual optic nerve atrophy (TCNR).
#10 [24] 1998	28; M; health ns; ME	Fever, drowsiness, nuchal rigidity.	OP: 21; CSF: WBC 95/μL (85% monos); prot 65; gluc 55	Given ceftriaxone, doxy. Outcome not described (TCNR).
#11 [24] 1998	25; M; health ns; ME	Fever, mild right hemiparesis.	OP: 20; CSF: WBC 97/μL (95% monos); prot 65; gluc 55	Given doxy, full recovery (RNR).
#12 [35] 1999.	79; sex and health ns; Encephalitis	Ataxia, intention tremor, adiadochokinesis.	EEG: diffuse cerebral involvement.	Outcome not specified (TCNR).
#13 [36] 2002	4, F, healthy, Meningitis	Eight days of fever, myalgias, rash. Given cefuroxime. Developed pharyngitis, conjunctivitis, cheilitis, lymphadenopathy, neck stiffness. Presumed Kawasaki Disease; given IVIG and aspirin.	CSF: WBC 22/μL (75% lymphs); prot 24; gluc ratio 0.52	Recovered (TCNR).
#14 [36] 2002	14, M, health ns; Meningitis	Six days of fever, chills, rash, and headache. Given cefatrizine. LP performed and penicillin was started.	CSF: WBC 20 (95% lymphs); prot 29; gluc ratio 0.46	+serology; given doxy for 10 days, recovered (RNR).
#15 [37] 2002.	50; M; healthy; Abducens n. palsy	Fever, arthromyalgia, asthenia, abdominal pain for one week; given doxy for 2 weeks; defervescence, normalized lab values; discharged.	Relapse: CT: normal. CSF: WBC 86/μL (75% lymphs); prot 100; gluc normal	One week after discharge, headache/diplopia; given cipro for 14 d; improved within 24 h (RNR).
#16 [38] 2003	22; M; healthy; Meningitis, facial n. palsy	Fever, headache for 7 d, mild nuchal rigidity, and left facial palsy; recently received amoxicillin for an earache.	CT: normal. OP: 23; CSF: WBC 136/μL (97% lymphs); prot 58; gluc ratio 0.4	Received ceftriaxone; after + serology, given doxy for 14 days; no comment on facial palsy resolution (TCNR).
#17 [39] 2004	Age, sex, health ns. Meningitis, renal failure	Dry cough and acute renal failure and later become disoriented. Conjunctivitis and rash appeared but waned shortly afterwards.	CT: normal. CSF: WBC 90/μL (monos); prot 70; gluc normal	Doxy was administered with rapid neurologic improvement (RNR).
#18 [39] 2004;	Age, sex, health ns; Meningitis	Presented with “progressive meningeal syndrome.”	CSF: WBC 19/μL (monos); prot 49; gluc normal	The patient completely recovered in 48 h after receiving doxy (RNR).
#19 [40] 2005	49; M; health ns; Encephalitis, seizure; cognitive impairment	Rash, bloody diarrhea, anemia, thrombocytopenia, hyponatremia. Patient had renal failure, seizures, coma; started acyclovir.	MRI: cortical swelling. CT (8 months after admission): encephalomalacia, limbic system atrophy	+ serology in week 2; given doxy; after 3 weeks, awoke from coma; severe cognitive impairment 8 months after presentation (PND).
#20 [41] 2005	18; M; health ns; ME	Meningitis, stupor, prostration.	no studies specified	Doxy × 10 days; outcome not described (TCNR).
#21 [42] 2007	57; F; healthy; Meningitis; retinitis	Five days of fever, headaches, and myalgias; persistent fever 72 h after hospitalization. Placed on ampicillin; remained febrile. Retinal hemorrhages, infiltrates, and vasculitis.	CSF: WBC 30/μL (78% lymphs); prot 48; gluc ratio 0.55	Given cipro for 7 days with fever resolution after 48 h (RNR).
#22 [43] 2010	19; F; health ns; Encephalitis, hemiparesis	Persistent fever, diarrhea, and HSM; penicillin started; developed left hemiparesis, anemia, and thrombocytopenia.	no studies specified	Doxy was started and after 4 days the patient became afebrile. Outcome not described (TCNR).
#23 [44] 2011	20; M; healthy; ME, bilateral abducens n. palsy	Presented with headache, nausea, vomiting, and paresthesias. Then he was unable to move or speak. Recovered fluent, but nonsensical, speech. Developed fever. Given ceftriaxone, ampicillin, acyclovir; then vancomycin, oseltamivir, and azithro.	OP: 30; CSF: WBC 180 lymphs/μL; prot 170; gluc normal	On day 10, diplopia and reduced vision due to abducens n. palsies and papilledema. Discharged day 17 after fever, headache resolved. Palsies resolved after 30 days (RAID)
#24 [27] 2014	17; F; healthy; ME with retinal, optic nerve hemorrhage and persistent cognitive impairment, facial n. palsy	Twelve-day history of fever, headaches, rash, vomiting, myalgia, and weakness. Diagnosed with viral illness and discharged. Fever resolved, headaches persisted. Eight weeks later, readmitted with vomiting headache, and diplopia. Discharged in 24 h. Then 1 week later; confusion, staring gaze, slurred speech, tremor, facial asymmetry, pronator drift; papilledema; retinal and optic nerve hemorrhage.	EEG: seizures. MRI brain: normal. CSF: WBC 184/μL; (97% lymphs); prot 83; gluc 49. EEG: left hemisphere slowing.	Diagnosed as viral ME. + serologic testing. Given doxy for 14 days. Two subsequent episodes of headaches, irritability, and slurred speech. Developed pseudotumor cerebri. One year after onset, patient had persistent memory and learning impairments (PND).
#25 [45] 2014	31, M, healthy; Abducens n. palsy	Two day history of fever, myalgias, and headache. After lumbar puncture, had unilateral abducens n. palsy. Fundoscopy one week after admission showed blurred optic disk margins. Doxy given × 1-day. Then levofloxacin × 7 days. + serologic test.	CT: normal. MRI: normal. OP: 23; CSF normal.	Abducens palsy started to resolve after 2 months and recovered completely 3 months after symptom onset (RAPD).
#26 [25] 2015	74; M; htn, alcohol abuse; Cerebral infarctions/hemorrhage	Fever, headaches for days, then impaired consciousness; started cefoperazone sulbactam and ribavirin; worsened to coma, stiffness, extensor plantar response. Second MRI: increased number of lesions; CT revealed subarachnoid hemorrhage.	First MRI: multiple lesions of brain stem, and cerebral/cerebellar hemispheres. CSF: WBC 20/μL; prot 57; gluc 52	Minocycline started. After 3 weeks improved, neurologic condition improved. Discharged to rehabilitation (RASD).
#27 [46] 2015	39; M; healthy; Meningitis, abducens n. palsy	Nine days of fever, night sweats, neck pain, diplopia, vomiting. Right lateral gaze palsy, papilledema.	MRI: increased pressure. OP: 39; CSF: WBC 26/μL (37% lymphs; 36% monos); prot 30; gluc 72.	Started on ceftriaxone, acyclovir, doxy; improvement of palsy 1 month after discharge (RAID).
#28 [46] 2015	27; M; health ns; ME with bilateral abducens n. palsy	Three days of fever, headache, then vomiting, progressive drowsiness. Intubated due to drowsiness. Started ceftriaxone, acyclovir, RIPE. Extubated after 2 weeks. Developed bilateral abducens palsy and dysmetria.	MRI: leptomeningeal enhancement, restricted diffusion in corpus callosum. EEG: no seizures. OP: 28; CSF: WBC 100/μL (89% lymphs); prot 40; gluc ratio 0.79	Hospitalized for 1 month, discharged to rehabilitation. + serology after discharge (TCNR).
#29 [18] 2018	46; M; alcohol abuse; fatty liver; Brain ischemia, multiorgan failure	Presented in status epilepticus. Intubated. Had acute kidney injury. Started vancomycin, cefepime, metronidazole, ampicillin, and acyclovir. Seizures despite anticonvulsants. Placed in a pentobarbital coma. Doxy started. Hemodialysis started.	CT: normal. OP: 17; CSF: WBC 66/μL (46% lymphs); prot 170; gluc normal	Due to progressive multi-organ failure, transitioned to comfort care and died on day 6. At autopsy, extensive necrosis of hippocampus, cerebrum (DEATH).
#30 [47] 2018	39; F; healthy; Abducens n. palsy	Five days of fever, headache, myalgias; started doxy; developed left abducens palsy.	MRI: negative.	Nerve palsy gradually improved (TCNR).
#31 [48] 2019	30; F; gastric bypass; ME, respiratory failure	Mental confusion and fever, later developed visual hallucinations. Cefepime and vancomycin given w/o improvement. Became hypoxic and hypotensive. Patient intubated.	LP: normal. MRI: leptomeningeal enhancement.	+ serologic test and doxy initiated, with rapid and complete resolution of symptoms (RNR).
#32 [49] 2019	31; M; healthy; ME, seizures	Ten days of fever, myalgias, headache; diagnosed as viral infection and discharged; returned 5 d later after seizures; started lorazepam, levetiracetam, ceftriaxone, ampicillin, vancomycin and acyclovir. Hemodialysis started for acute kidney injury.	CT: normal. CSF: WBC 126 cells/μL (80% pmns); prot 99; gluc 56	Depressed consciousness; intubated. Doxy added. Neuro status improved over 5 days; extubated; improved renal function; full recovery (RNR).
#33 [50] 2020	39; M; health ns; Meningitis	Fever, malaise, myalgias, Took 5 d of azithro; no improvement; on day 10 of fever went to ER; discharged on analgesics. Returned to ER 2 d later with headache, fever.	CT: normal. CSF: WBC 28/μL (52% lymphs; 27% pmn); prot 40; gluc ratio 0.35	Ceftriaxone given, fever and lethargy persisted. LP done; added acyclovir; + serology; given 14 d of doxy with full recovery (RNR).
#34 [51] 2021	18; F; healthy; Abducens n. palsy	Fever, received FQ; then headache, diplopia; on examination, optic disk swelling, small retinal infiltrate with retinal hemorrhage.	MRI brain and orbit: normal.	Received doxy for 15 d; resolution of diplopia, retinal changes (RNR).
#35 [52] 2023.	75; M; mild dementia; ME w/Parkinsonism and normal pressure hydrocephalus	Presented with confusion, shuffling gait, imbalance, rigidity, facial grimacing, irritability, insomnia. Oriented to person only. Then he developed fever and rash. A presumptive diagnosis of FBT was made and doxy was given for 10 days. + serologic testing. Developed urinary incontinence and LP repeated; 300 cc CSF removed. Gait improved.	LP #1: CSF WBC 1/µL; prot 53; gluc 68. MRI: volume loss and ischemic changes (likely baseline). LP #2: CSF: WBC 2/µL; prot 47; gluc 65	Pt displayed mixed hypoactive/hyperactive delirium. After 9 doses of doxy, he returned to his cognitive baseline. He was discharged to a memory care facility (RNR).
#36 [53] 2024	4: M; healthy; Encephalopathy	Three days of fever, altered mental status, unable to bear weight on right leg; CK elevated. Given ceftriaxone, vancomycin, dexamethasone.	CT: normal. CSF: WBC 0; prot 14; gluc 64	Altered mental status resolved within 12 h. Given doxy; within 24 h fevers trended down and hip pain improved (RNR).
#37 [54] 2024	76; M; health ns; ME, seizures	Fever, chills, nausea, headache; given vancomycin, ceftriaxone, acyclovir, ampicillin, pip-tazo, cefepime; pt had seizures.	CT: normal. CSF: WBC 27/μL (41% lymphs); prot 67; gluc ratio 0.5	Given doxy for 12 days; died after a 26-day delay in appropriate recognition and treatment (DEATH).
#38 [54] 2024	42; M; health ns; ME	Fourteen days of fever, headache, malaise; given acyclovir, ceftriaxone, vancomycin.	CT: Hyperdense left frontal lobe lesions. CSF: WBC 16/μL (50% pmns); prot 50; gluc ratio 0.48	Doxy, 21 days; survived, chronic headaches (TCNR).
#39 [54] 2024	22; M; health ns; Meningitis	Ten days of fever, headache, malaise, vomiting; given ceftriaxone, azithro.	CSF: WBC 77/μL (76% lymphs); prot 44; gluc ratio 0.55	Doxy, 21 days; recovery, no sequelae (RASD).
#40 [54] 2024	30; M; health ns; Meningitis	Fever, chills, headache, myalgias, nausea, vomiting, blurry vision, rash; given vancomycin, pip-tazo, dexamethasone.	CSF: WBC 30/μL (100% monos); prot 24; gluc ratio 0.48	Given doxy for 5 days; lost to follow-up (TCNR).
#41 [55] 2025	49; F; health ns; Meningitis	Day 1: fever, cough, vomiting, diarrhea; Day 2: cervicalgia, headache, rash; given pip-tazo.	CSF: WBC 13/μL (75% pmn); prot 55; gluc ratio 0.55	+ serology; given 21 days of doxy; survived, chronic headaches (TCNR).
#42 [55] 2025	39; F; health ns, Meningitis	Day 1: headache, weakness, fever, myalgias, neck pain; Day 2: ocular pain; given azithro, amoxicillin-clavulanic acid, clindamycin.	CSF: WBC: 92/μL (44% pmns); prot 62; gluc ratio 0.56	+ serology; given 21 days of doxy; recovered, chronic headaches (TCNR).
#43 [56] 2025	66; M; alcohol abuse, cirrhosis; Encephalitis	Progressive confusion, anorexia, fever. On examination, lethargic, disoriented, in atrial fibrillation. Severe hyponatremia (119 mEq/L). Had myocardial infarction. Pip-tazo and vanco started.	CT scan: normal. MRI: normal.	+ serology. Doxy started; pip-tazo and vanco stopped. Ceftriaxone and ampicillin started. Mental status did not improve until doxy was started (RNR).
#44 [this paper]	40; M; epilepsy; recent travel to Panama and Colombia; ME, seizures	Fever, chills, cough, headaches, diarrhea; given azithro, prednisone. Fevers continued, went to ER; had seizures and required intubation. Given levetiracetam, valproic acid, and lacosamide. Fevers continued. Started on acyclovir, doxy, vancomycin, ceftriaxone, and artemether–lumefantrine (A-L). Fever improved by day-4 and kidney injury and encephalopathy resolved.	EEG (after anticonvulsants): stupor. MRI: normal. First LP: OP: 37; CSF WBC 276/μL (68% pmns, 17% lymphs, 12% monos); prot 112; gluc 36	A-L was stopped after malaria smears were negative. Repeat LP on day-4 showed decreased OP and improved cell counts (Table 1). He was extubated, following which he was alert and oriented, with persistent headaches. + serology. Discharged on doxy for 10 days. Two weeks later, he was asymptomatic (RASD).
#45 [this paper]	68; M; htn, heart failure, gout, type 2 diabetes; Encephalitis	Two days of headaches, fatigue, myalgias, and fever; somnolent but arousable. On hospital day 5, worsening somnolence, pulmonary edema, atrial fibrillation, and hemodynamic instability; required intubation, vasopressors, and dialysis. + serology.	CT: diffuse cerebral edema. MRI: restricted diffusion of pons. CSF: WBC 3/μL; prot 67; gluc ratio 0.60	Given doxy for 14 days. Remained obtunded for two weeks. Weaned from mechanical ventilation. At discharge, able to follow commands. Two months later, recovered except mild residual cognitive impairment (RAPD).

^a^ Abbreviations: azithro, azithromycin; chloramp, chloramphenicol; cipro, ciprofloxacin; CSF: cerebrospinal fluid; CT, computerized tomography; doxy, doxycycline; EEG, electroencephalogram; ER, emergency room; gluc, glucose (mg/dL); gluc ratio, [CSF glucose]/[serum glucose]; HSM, hepatosplenomegaly; htn, hypertension; IVIG, intravenous immunoglobulin; LP, lumbar puncture; lymphs, lymphocytes; monos, monocytes; MRI, magnetic resonance imaging; ns, not specified; OP, opening pressure of the lumbar puncture (cm H_2_O); pip-tazo, piperacillin-tazobactam; PCN, penicillin; prot, protein (mg/dL); RIPE, rifampin-isoniazid-ethambutol-pyrazinamide; prot, protein (mg/dL); WBC, white blood cells; TMP-SMX, trimethoprim-sulfamethoxazole; W-Ft, Weil–Felix test.

### 4.2. Review of Cases of Neurologic Presentation of Flea-Borne Typhus

The characteristics of the two cases reported herein and 43 other cases of FBT with CNS manifestations from the literature are summarized in Table 2. As in all analyses based on the retrospective reporting of individual cases, there may be a publication bias in which more exceptional cases were more likely to be published. To categorize the time course of the outcomes of the cases, we divided the cases into seven groups: rapid neurologic recovery (RNR) (<2 weeks); recovery after short duration (RASD) (>2 weeks <1 month); recovery after intermediate duration (RAID) (1–3 months); recovery after prolonged duration (RAPD) (>3 months, <6 months); persistent neurologic deficits (PND) (>6 months); death; and time course not reported (TCNR). Any known outcome other than RNR was considered delayed neurological recovery in the statistical analyses below.

The first entry in Table 2 was identified as a case of FBT meningitis by Woo and coworkers. However, this case was excluded from our analysis because the high level of CSF protein and the high neutrophilic pleocytosis are inconsistent with the other cases. Also, the diagnosis of FBT in the Woo case [32] was based on the Weil–Felix test, which has low specificity [57]. The second entry in Table 2, #Jang, was also excluded from the analyses because a brain abscess is inconsistent with typical typhus pathogenesis; rickettsial infection has not been previously reported to cause abscesses. Furthermore, the serologic findings in this case, a positive IgM and a negative IgG, are inconsistent with a course of FBT that extended over months [33]. Including our two cases described herein, 45 cases of FBT with neurological involvement with case details were identified. The ages of the patients were specified in 43 cases, with a mean age of 37.4 and a range of 4–79 years. The mean age of the 40 adult patients was 39.7 years (additional statistics on the age composition are provided in Table S1 of the Supplemental Materials). Of the 39 adult patients of Table 2 with a specified age, 26/39 were male (66.7%) and 13/39 (33.3%) were female. The preponderance of male patients compared to females was statistically significant (χ^2^ (1, N = 40) = 4.90, *p* = 0.027). The observed male predominance may reflect reporting bias, flea exposure differences between males and females, or other unidentified factors. In a summary of case series of 2074 FBT patients, there was also a male predominance [1].

The final diagnoses were meningoencephalitis (16 cases), meningitis (14 cases), encephalitis (9 cases), isolated nerve palsies (3 cases), brain infarctions (2 cases), and meningoencephalitis with Parkinsonism (1 case). Seizures occurred in seven patients, including Case Report #1. Abducens nerve palsy was seen in eight cases (two of which were bilateral). Abducens nerve palsy is the most commonly observed oculomotor cranial neuropathy; in FBT, abducens nerve palsy likely arises from increased intracranial pressure or vasculitis of the blood vessels supplying the nerve. In bilateral abducens nerve palsies, additional possible etiologies include intracranial hemorrhage, subdural hematoma, and brainstem infarction [58], but these more severe etiologies were not apparent in this series. Two patients had facial nerve palsies and four had optic nerve involvement. Although increased CSF pressure may contribute to optic disk edema, vasculitis has also been proposed as an etiology in FBT [11].

An opening pressure (OP) of the lumbar puncture was reported for 13 adult patients (Table 2); the median initial OP was 23.0 cm H_2_O (range 5–39 cm H_2_O). Only three studies reported an initial OP < 20 cm H_2_O. Thirty-three patients had lumbar punctures performed that displayed at least one CSF abnormality (pleocytosis, elevated protein levels, and/or decreased glucose levels). The median number of white blood cells in the initial abnormal CSF specimens was 30 cells/μL (range 1–321/μL). In most cases, the cellular infiltrate was predominately lymphocytic. In two cases, the initial LP revealed a neutrophilic pleocytosis, which converted to lymphocytic in subsequent lumbar punctures. Of the adult patients with abnormal CSF, 26/33 (78.8%) had elevated protein levels; the median value was 60 mg/dL (range 24–185 mg/dL) (for detailed statistics on the OP and CSF WBC and protein, see Table S2 in the Supplemental Materials). The CSF of 11 adult patients displayed hypoglycorrhachia (11/33, 33.3%), including Case Report patient #1. We wanted to determine if there are any correlations between the lumbar puncture and cerebrospinal fluid parameters of opening pressure, pleocytosis, elevated protein levels, and the presence or absence of hypoglycorrhachia. However, no statistically significant correlations between these parameters were found (Table 3), but these calculations were underpowered.

Of the 31 patients with a documented time course of recovery (or death), 18 experienced rapid neurologic recovery (RNR; 18/31, 58.1%), three had recovery after a short duration (RASD; 3/31, 9.7%), three after an intermediate duration (RAID; 3/31, 9.7%), two after a prolonged duration (RAPD; 2/31, 6.5%), and two had persistent neurologic deficits (PND; 2/31, 6.5%). There were three deaths among the 45 patients (6.7%), which is much higher than the 0.33% overall death rate typically observed in FBT in the post-tetracycline era [1]. The three deaths occurred in adult patients, so in this group the death rate was 3/42 (7.1%). One patient (#4) died of cardiac arrest [11]. One elderly patient (#37) died despite receiving doxycycline, presumably due to a delay in making the appropriate diagnosis and initiating doxycycline [54]. In the third fatal case (#29), the patient presented in status epilepticus, quickly progressed to multiorgan failure, and was found to have extensive necrosis of the cerebral hemispheres and hippocampus at autopsy [18]. We analyzed the lumbar puncture and CSF parameters of OP, WBC, and protein level with respect to the course of neurologic involvement (rapid neurologic recovery (RNR) versus delayed neurologic recovery (RASD, RAID, RAPD, PND, or death) and found no statistically significant relationships (Table 3). The sex of the adult patients was also found to not be statistically associated with neurologic outcome (χ^2^ (1, N = 29) = 0.9, *p* = 0.34). Furthermore, among the adult patients, older age was also not statistically associated with delayed neurologic recovery (point biserial (r_pb_ (27) = −0.062, *p* = 0.75)). However, these interpretations regarding age and sex and outcome are limited by the retrospective nature of the current study, small sample sizes, and incomplete and heterogenous reporting in the original publications.

A CT of the head was done near the time of admission in 14 cases (14/45, 31.1%). It was abnormal in two of the 14 cases (2/14, 14.3%); one case showed diffuse cerebral edema (our Case Report #2) and the other displayed hyperdense frontal lobe lesions. An MRI brain was performed near the time of admission in 13 cases (13/45, 28.9%). Seven of the 13 MRI scans (53.8%) were abnormal; findings ascribed to FBT included cortical swelling, infarctions, leptomeningeal enhancement, and restricted diffusion. However, the MRI scan was normal in 6/13 studies (46%), including in Case Report patient #1. Visualization of the glial nodules, a common histopathologic feature of neurologic involvement in rickettsial infections, by magnetic resonance imaging is not possible, as these are microscopic findings. This normal appearance of the MRI in some cases is likely due to the relatively low level of meningeal inflammation observed in many cases of FBT (based on cell counts and protein levels) as compared to pyogenic bacterial meningitis [59]. The cerebral edema observed in Case Report #2 parallels prior autopsy descriptions of diffuse cerebral swelling in severe FBT; the restricted diffusion revealed in the MRI of Case Report #2 was also seen in the MRI of case #28 in Table 2.

Forty-one patients (41/45, 91.1%) received an antibiotic with potential anti-rickettsial activity (a tetracycline, chloramphenicol, azithromycin, ciprofloxacin, or levofloxacin). Thirty-eight patients (38/45, 84.4%) were treated with a tetracycline (doxycycline, 35; tetracycline, 2; minocycline, 1). Several papers commented on the rapid improvement that occurred once a tetracycline was initiated. However, two of the patients that died also received doxycycline, but likely the initiation was too late in the course of the illness. One patient relapsed after a course of doxycycline, but the infection resolved after ciprofloxacin treatment. Two patients were given chloramphenicol as primary therapy; one relapsed after chloramphenicol was stopped, and the other had a slow recovery over two months. In the treatment of rickettsioses, chloramphenicol is associated with a longer period to defervescence and higher rates of relapse and death as compared to therapy with a tetracycline [60]. Five patients received initial azithromycin as empiric therapy for fever; in four cases, it was deemed to be inadequate therapy and the patients were also given doxycycline.

The most complete assessment of FBT CNS infection from a single cohort derives from a 2015 study conducted in Laos of 1112 adult and pediatric patients admitted with a neurological presentation that received a lumbar puncture [59]. Twenty-eight patients with a neurologic presentation of FBT (NPFBT) were identified; headache, stiff neck, and seizures were present in 84, 69, and 25% of patients, respectively. Hearing loss and photophobia were not observed. Meningitis was diagnosed in 68% of the NPFBT cases, and meningitis with encephalitis was seen in 57%. The median opening pressure was 17 cm H_2_O with a range of 9–40 cm H_2_O. The median CSF leukocyte count was 10/mL (range 0–605/mL), with an average neutrophil to lymphocyte ratio of 1. CSF protein and lactate levels were elevated in 46% and 33% of NPFBT patients, respectively, with hypoglycorrhachia seen in 38%. The mortality rate in NPFBT was 27%, higher than in neurologic presentations of scrub typhus or leptospirosis (14 and 13%, respectively), but less than in pyogenic meningitis (33%). However, these mortality differences were not statistically significant. There was no assessment of the association between mortality and neurologic/CSF characteristics or concurrent dysfunction of other organs [59]. Thus, compared to our series of 45 patients (Table 2), the Laotian cases had a lower median OP of the lumbar puncture (17 vs. 23.0 cm H_2_O), a lower median level of pleocytosis (10 vs. 48.0 cells/μL), and a lower percentage patients with elevated CSF protein levels and hypoglycorrhachia (46% vs. 78.8% and 24% vs. 33.3%), respectively. The Laotian series patients with NPFBT also had a higher mortality rate than the patients reported in Table 2 (27% versus 6.7%), but this may be due to a lower level of supportive medical care available in Laos than in the countries of residence of the case patients presented in Table 2.

In another Laotian series of 66 patients with severe neurological infections, nine had bacterial meningitis (BM), 11 had tuberculous meningitis (TBM), 25 had Japanese encephalitis (JE), and 21 had rickettsial infections (including 11 with scrub typhus). Seizures were more common in rickettsial CNS infection than in bacterial or tuberculous CNS infection, but not as common as in JE [28]. Patients with rickettsial CNS infections showed the astrocyte activation markers glial fibrillary acidic protein (GFAP) and S100b within or around the normal range compared with the elevated levels seen in TBM or BM cases. Patients with rickettsial CNS infections had higher median levels of the CSF tau protein (a phosphoprotein that binds tubulin and promotes microtubule assembly and stability) than BM cases, suggestive of neuronal/axonal damage, but not as high as in JE. In general, patients with TBM and BM had significantly higher blood–brain barrier leakage and greater inflammatory responses in the CSF with raised lactate, leukocytosis, protein release, and decreased CSF/blood glucose ratio than those with CNS infection due to either FBT or scrub typhus [28]. In a Texas series of eight fatal FBT cases, neurologic complications (meningitis, encephalitis, vertigo, dizziness, seizure, and coma) were evident in five of the deceased patients (62.5%) [61]. Neurologic complications of FBT are also more common in elderly patients [62].

### 4.3. Cytologic Exam of the Cerebrospinal Fluid

Plasmacytoid lymphocytes and plasma cells were seen on CSF cytologic exam of case patient #1. The plasmacytoid lymphocyte is an activated B lymphocyte in the process of transforming into a plasma cell. The nucleus is eccentric, the chromatin is more clumped, and the cytoplasm is more basophilic than in a typical lymphocyte [63]. Plasmacytoid lymphocytes in the CSF have been previously described in various viral infections, Lyme disease, tuberculosis, and syphilis [64]. However, to our knowledge, these findings have not been previously described in neurologic rickettsial infections caused by members of the order Rickettsiales (major genera *Rickettsia*, *Anaplasma*, *Orientia*, and *Ehrlichia*). Cerebrospinal fluid plasma cell pleocytosis has been observed in viral meningoencephalitis (West Nile virus, enterovirus), neuroborreliosis, tuberculous meningitis, neurosyphilis, zoster meningoencephalitis, HIV infection, neurocysticercosis, and African trypanosomiasis [65], but, to our knowledge, has not been reported in rickettsial infections. The recruitment of potential antibody-secreting cells, such as plasmacytoid lymphocytes and plasma cells, into the CSF is part of the immunologic response to pathogen invasion of the CNS. However, it is important to bear in mind that the specific morphology of the cells in the CSF during the course of a CNS infection may vary over time [66].

### 4.4. Approximate Incidence of CNS Involvement in Flea-Borne Typhus

To determine an approximate incidence of FBT CNS disease, we examined 31 case series, which included 2508 patients with FBT (Table 4). A limitation of this analysis is that these case series extend from 1945 to 2018; the present-day brain imaging techniques and modern serologic methods were not available for series conducted in these earlier times periods, leading to an uncertain level of missed and/or erroneous diagnoses. Thus, these estimates of neurologic manifestations and complications should be interpreted cautiously given heterogeneity in study design, diagnostic criteria, reporting practices, and severity thresholds across the various case series. It is generally accepted that pediatric patients have a milder course of FBT than adult or elderly patients [1]. However, 17 of the case series included both pediatric and adult patients, and the age ranges dividing pediatric from adult patients overlapped between many of the series. Thus, it is difficult to compare the pediatric and adult data.

In Table 4, the diagnostic categories may overlap in some cases. For example, a patient with meningitis may also present with lethargy; this overlap will cause an overestimation of the overall occurrence of neurologic involvement. For the pediatric data, P1–P6, altered sensorium (stupor, lethargy, and confusion) was relatively common (6.3%), whereas meningitis occurred in only about 2% of patients, and seizures and ataxia both occurred very rarely. For the adults only data (A1–A7), altered sensorium occurred in 2.8% and seizures in only 0.4%. No cases of meningitis were described. In the combined group of both pediatric and adult patients, altered sensorium occurred in about 4% of patients, meningitis was seen in less than 1%, and ataxia and cerebellitis were very rare. When all 2508 patients with FBT are considered, about 5% had altered sensorium during their illness, and meningitis and seizures occurred in only 1% and 0.12%, respectively. Thus, overall, CNS manifestations of FBT are uncommon, especially more severe presentations, such as meningitis, meningoencephalitis, and cranial nerve palsies, as described in many of the cases of Table 2. However, in the study of 49 elderly patients [62], altered sensorium occurred in 22.5% of patients, and meningitis was documented in 10.2% of patients. Thus, for the elderly only group, altered sensorium and meningitis did appear to be more common than in the overall group (which also contained an unspecified number of elderly persons). Meningitis was also described at a higher rate in the pediatric group (2%) compared to the adults only group (0%). There was a significant difference in the rate of meningitis in the elderly patients (10.2%) compared to pediatric patients (1.9%) (χ^2^ (1, N = 527) = 11.9, *p* < 0.001). Although reported meningitis rates appeared higher in the elderly cohort, this interpretation is limited by differences in study design and diagnostic evaluation. Compared to FBT, neurologic involvement occurs much more commonly in RMSF and scrub typhus (20–33% and 25–46% of cases, respectively) [11,95]. The microbial etiologies, vectors, neurologic manifestations, and mortality rates of FBT, RMSF, and scrub typhus are summarized in Table 5. From Table 5, it is evident that RMSF and scrub typhus are more severe infections than FBT based on the mortality rates in both the pre- and post-antibiotic periods. The CNS vasculitis is more severe in RMSF than in the other two infections. Scrub typhus is more likely to have immune-mediated pathological effects in the nervous system. Movement disorders are also more common in scrub typhus, including the opsoclonus-myoclonus-ataxia syndrome, which has not been reported in FBT and RMSF. Patients stricken with RMSF are more likely to suffer severe neurologic sequelae.

### 4.5. Considerations in the Treatment of Neurologic Presentations of Flea-Borne Typhus

The severe neurologic manifestations observed in several patients of Table 2, including prolonged encephalopathy, cerebral edema, persistent neurologic deficits, and death, raise the issue of whether optimized tetracycline class drug dosing strategies merit further investigation. All of these treatment proposals described below are based on biological plausibility and prior observations in other infections and would need to be proven in clinical trials for use in FBT. Although doxycycline at the standard dosing of 100 mg twice a day is considered the treatment of choice for rickettsial infections, a consideration of the pharmacologic properties of drugs in the tetracycline class suggests other treatment strategies should be explored in patients with severe manifestations of FBT and other rickettsial infections. In critically ill patients with scrub typhus, a 200 mg intravenous loading dose of doxycycline has been utilized because of the altered volume of distribution in such patients [110]. In fact, for the newer tetracycline drugs tigecycline and omadacycline, a loading dose is on the label of the drugs. Another problem is that doxycycline has poor CNS penetration, achieving only 14% of the serum concentration [111]. To enhance doxycycline levels in the CNS, 200 mg twice daily has been proposed. Nevertheless, despite its modest CSF penetration, doxycycline remains highly effective in most rickettsial infections, likely because the principal cellular targets of *R. typhi* and other rickettsiae are vascular endothelial cells [17,23]. Even in the CNS, the apical membranes of endothelial cells are in direct contact with blood, thus reducing concerns for delivering therapy to the site of infection. Minocycline has also been advocated over doxycycline for the treatment of CNS infections because of its superior CNS penetration [112]. Another consideration is the use of corticosteroids in patients with neurologic presentations of FBT, because these are beneficial in the treatment of pyogenic and tuberculous meningitis [113]. Whether adjunctive corticosteroids could mitigate inflammatory CNS injury in severe neurologic FBT remains unknown. No current guidelines recommend the use of higher doses of doxycycline or minocycline or the use of corticosteroids to treat rickettsial infections of the central nervous system.

## 5. Conclusions

The two cases described herein and the 43 cases abstracted from the literature show that patients with neurologic involvement in FBT may suffer serious short-term and long-term neurologic consequences, including death. These patients may have prolonged hospitalization and may require intubation due to their neurologic impairment or seizures. In an FBT-endemic area, FBT should be considered in the differential diagnosis of patients who present with a meningitis or encephalitis-like illness. Some of these patients may be so neurologically impaired on presentation that it may not be possible to obtain a history that may reveal epidemiologic clues to the diagnosis. Although many patients recover rapidly with appropriate antibiotic treatment, neurologic involvement in FBT may result in prolonged recovery, persistent neurologic deficits, or death. It is likely that prompt initiation of doxycycline in cases of neurologic presentation of FBT may reduce the morbidity and mortality associated with this affliction.

## Figures and Tables

**Table 1:** Cerebrospinal fluid (CSF) analyses of case patient #1.

Parameter	Day 1	Day 4	Reference Range	Units
Opening pressure	37	23.5	7–18	cm H_2_O
Total nucleated cells	276	33	≤5	cells/μL
Neutrophils (%)	68	0		
Lymphocytes (%)	17	86		
Monocytes (%)	12	14		
Red blood cells	37	6	0–5	cells/μL
Protein	112	59	15–45	mg/dL
Glucose	36	50	45–75	mg/dL

**Table 3:** Correlations of opening pressure, CSF parameters, and outcome ^a^.

Entry	Parameter 1	Parameter 2	*n*	Test	Correlation	*p*
1	WBC	OP	13	Spearman’s rho	0.46	0.14
2	WBC	Protein	31	Spearman’s rho	0.28	0.13
3	WBC	+/− Hypoglycorrhachia	30	Rank Biserial	0.06	0.81
4	WBC	Delayed Neurorecovery	22	Rank Biserial	0.13	0.64
5	OP	Protein	12	Spearman’s rho	−0.32	0.31
6	OP	+/− Hypoglycorrhachia	11	Point Biserial	0.01	0.98
7	OP	Delayed Neurorecovery	8	Point Biserial	0.56	0.15
8	Protein	+/− Hypoglycorrhachia	30	Rank Biserial	0.02	0.95
9	Protein	Delayed Neurorecovery	22	Rank Biserial	0.30	0.25

^a^ WBC and protein levels were found to have a skewed distribution, but OP values were normally dis-tributed.

**Table 4:** The incidence of CNS disease in case series of flea-borne typhus patients: pediatric (P1–P6), adult (A1–A7), elderly (E), and pediatric + adult (PA1–PA17).

Series	[Ref], Publication Year	Location	Number of Patients	Age Range (Years)	CNS Manifestation(s): Number (Percent)
P1	[67], 2000	USA	30	2–17	Meningitis: 1 (3.3%)
P2	[68], 2001	USA	97	5 months–16	Stupor: 5 (5.2%) Ataxia: 1 (1.0%)
P3	[69], 2006	Israel	76	Children	0
P4	[70], 2007	Cyprus	21	4–13	Meningitis: 1 (4.8%)
P5	[71], 2009	Greece	41	1–15	Confusion, normal CSF: 1 (2.4%)
P6	[72], 2018	USA	213	3 months–19	Lethargy: 24 (11.4%) Seizures: 1 (4.7%) Meningitis: 7 (3.3%)
**TOTAL PEDIATRIC PATIENTS:**			478	**TOTALS:**	Meningitis: 9 (1.9%) Seizures: 1 (0.2%) Stupor, lethargy, confusion: 30 (6.3%) Ataxia: 1 (0.2%)
A1	[73], 1988	Thailand	170	Adults	0
A2	[74], 2001	Singapore	21	Mean age 38.2	0
A3	[75], 2003	Spain	32	Adults 18–73	0
A4	[76], 2006	Tunisia	11	16–79	0
A5	[77], 2007	New Zealand	12	Adults	0
A6	[78], 2012	Greece	90	18–89	Stupor: 8 (8.9%) Coma: 2 (2.2%)
A7	[79], 2015	Vietnam	193	16 and older	Altered mental status: 4 (2.1%)
**TOTAL ADULT PATIENTS**			529	**TOTALS:**	Altered mental status: 4 (0.8%) Stupor: 8 (1.5%) Coma: 2 (0.4%) Seizures: 2 (0.4%)
E	[62], 2014	Greece	49	66–90	CNS disorders: 10 (20.4%) Stupor: 6 (12.3%) Cognitive disorders: 3 (6.1%) Coma: 1 (4.1%) Meningitis: 5 (10.2%) (unspecified overlap with stupor, cognitive disorders, and coma)
PA1	[80], 1945	USA	180	20 months–67	Stupor: 28 (15.6%) Delirium: 12 (6.7%) Coma: 3 (1.7%)
PA2	[81], 1946	USA	126	24 children, 102 adults	Meningitis: 3 (2.4%)
PA3	[82], 1986	USA	200	1–90	0
PA4	[83], 1991	USA	80	Children, adults	Confusion, stupor, coma, or hallucinations: 10 (12.5%) Seizure: 3 (0.8%) Ataxia: 1 (1.3%)
PA5	[84], 1992	Greece	49	7 children (0–19); 42 adults (19–79)	0
PA6	[35], 1999	Spain	104	12–81	Cerebellitis: 1 (1%)
PA7	[85], 2002	Greece	83	14–80	Confusion: 8 (9.6%) Unspecified CNS involvement: 8 (9.6%)
PA8	[39], 2004	Spain	22	14–76	Meningitis: 2 (%)
PA9	[86], 2008	Colombia	13	5 to 73	0
PA10	[87], 2008	Nepal	50	15–85	0
PA11	[88], 2010	USA	33	7–64	Confusion: 4 (12%)
PA12	[89], 2012	Taiwan	81	10–86	Meningitis: 3 (3.7%)
PA13	[90], 2012	Travelers	32	1–69	0
PA14	[91], 2012	Cyprus	193	3–89	0
PA15	[92], 2013	Tunisia	43	8–83	0
PA16	[93], 2015	Tunisia	73	13–68	Meningitis: 2 (2.7%)
PA17	[94], 2017	USA	90	Children, adults	Meningitis: 1 (1.1%)
**TOTAL PEDIATRIC + ADULT** **PATIENTS:**			1452	**TOTALS:**	Meningitis: 11 (0.8%) Altered sensorium: 62 (4.3%) Cerebellitis: 1 (0.07%) Ataxia: 1 (0.07%)
**GRAND TOTAL** **(P + A + E + PA)**			2508	**TOTALS:**	Meningitis: 25 (1.0%) Altered sensorium: 116 (4.6%) Seizures: 3 (0.12%)
**GRAND TOTAL** **excluding** **elderly series E**			2459	**TOTALS:**	Meningitis: 20 (0.8%) Altered sensorium: 106 (4.3%) Seizures: 3 (0.12%)

**Table 5:** A comparison of flea-borne typhus, Rocky Mountain spotted fever, and scrub typhus.

	FBT	RMSF	Scrub Typhus
Pathogen	*R. typhi*	*R. rickettsii*	*Orientia tsutsugamushi*
Major Vectors	Various fleas	Various hard ticks	Mites of genus Leptotrombidium.
Rash	No inoculation eschar [96]; rash occurs in 54–63% of patients; begins on thorax; face, palms, soles likely spared [97]. Macular: 49%; maculopapular: 29%; papular 14%; petechial: 6%; morbilliform: 3% [83].	Rare inoculation eschar [98]. Rash begins on extremities, more likely to be hemorrhagic; more likely to involve face, palms, and soles that in FBT [97].	Inoculation eschar in 7–80% of cases. The maculopapular rash starts on the trunk and then spreads to the limbs [99].
Death rate in pre-antibiotic era	3.6–7.6% [100,101]	25% [98]	70% [102].
Death rate in post-antibiotic era	0.33% [1]	3% [103]	Inpatients: 5.0–8.1% [104].
CNS Histopathologic findings (all three infections may display typhus nodules in the brain)	The vasculitis usually does not extend to all layers of the blood vessel [18]	The vasculitis may extend to all layers of the blood vessel, leading to necrosis of the intima and the media, with thrombotic occlusion and microinfarcts [105].	Vasculitis and peri-vasculitis due to proliferation of *O. tsutsugamushi* in endothelial cells. Secondary immune-mediated mechanisms and hemorrhage and infarction may contribute to pathogenesis [99,106].
Rate of neurologic presentation	6.7% (this paper)	20–33% [11]	25–46% [95].
Course of encephalitis/manifestations	Not enough cases to generalize course of neurologic involvement; Manifestations (this paper); Meningoencephalitis: 37.7% Meningitis: 31.1% Encephalitis: 20% Brain infarctions: 4.4% Seizures: 15.6% Abducens n. palsy: 17.8% Facial n. palsy: 4.4% Optic neuritis: 8.8%	Manifests first as confusion or lethargy in 26% to 28% of cases. With progression, stupor or delirium is seen in 21–26%, ataxia in 18%, coma in 9–10%, and seizures in 8%. Coma or seizures are associated with a fatal outcome [107].	Neurologic manifestations start in the second week of illness [99,106]. Meningitis (26% of all scrub typhus cases), Meningoencephalitis, Encephalitis, Seizures (6–21% of all cases), Cranial nerve palsies (5–10% of all cases), Cerebrovascular events (<1% of all cases), Movement disorders (5% of all cases), Cerebellitis, Trigeminal neuralgia, Demyelinating neuropathy, Neuropathy/radiculopathy, Transverse myelitis (<5% of all cases), Opsoclonus-myoclonus-ataxia syndrome.
Rate of long-term neurologic sequelae in patients with CNS involvement	6.5% (this paper)	More than half of patients have persistent neurologic deficits more than one year after the illness [105].	In 146 children with acute encephalitis due to scrub typhus, 62.3% had mild, 10.3% had moderate, and 4.7% had severe disability [108].
Long-term neurologic sequelae	Cognitive impairment (this paper)	Cognitive impairment; paraparesis; hearing loss; blindness; peripheral neuropathy; bowel and bladder incontinence; cerebellar, vestibular, and motor dysfunction; and speech disorders [98]	Long-term neurologic prognosis is good with treatment [99]. Some children may suffer persistent seizures and focal neurological deficits [109].

## Data Availability

The original contributions presented in this study are included in the article/Supplementary Materials. Further inquiries can be directed to the corresponding author.

## Supplementary Materials

The following supporting information can be downloaded at: https://www.mdpi.com/article/10.3390/pathogens15060590/s1, Negative Tests for Case Report #1, Table S1: Age statistics for the patients in Table 2; Table S2: Detailed statistics of the opening pressure, CSF WBC counts, and CSF protein levels for the patients in Table 2.
