# Supplementary material for: Meningoencephalitis in Flea-Borne Typhus: A Report of Two Cases and a Review of CNS Complications
Source: Pathogens. 2026 May 30;15(6):590. doi: 10.3390/pathogens15060590 (PMC13304721; doi:10.3390/pathogens15060590)
Supplement: Supplementary file 1 [file pathogens-15-00590-s001.zip › pathogens-4299719-supplementary.pdf]

Negative Tests for Case Report #1

Negative tests included: urine drug screen; human immunodeficiency virus antigen/antibody assay and PCR; hepatitis A, B, and C antibody screen; syphilis screen, chlamydia and gonorrhea triple screen (urine, rectal, pharyngeal); three malaria thick smears performed on separate days; an interferon-gamma release assay for tuberculosis (QuantiFERON TB Gold Plus (QIAGEN, Germantown, MD)), *Legionella* urine antigen, and two sets of blood cultures. Dengue fever virus antibody, yellow fever IgM by enzyme-linked immunosorbent assay (ELISA), total *Brucella* antibody by agglutination, *Bartonella henselae* and *Bartonella quintana* IgM and IgG were also negative. West Nile Virus IgM and IgG by ELISA and an autoimmune encephalitis panel of the CSF were also negative.

Table S1. Age statistics for the patients in Table 2.

	Age, years
Range (all patients, N=43)	4-79
Mean (all patients)	37.4 (*SD* 20.0)
Range (adults, N=40)	17-76
Mean (adult)	39.7 (SD 18.9)
Median (Adult)	36.5 (Q1-Q3 23.5-49.5)

Table S2. Detailed statistics of the opening pressure, CSF WBC counts, and CSF protein levels for the patients in Table 2

	Opening pressure, cm H_2O (N = 13)	CSF WBC, cells/µL (N = 33)	CSF protein, mg/dL (N = 33)
Mean	23.9 (*SD* 9.3)	71.7 (*SD* 76.8)	71.0 (*SD* 40.6)
Median	23.0 (*Q1-Q3* 18.5-30.5)	30 (*Q1-Q3* 19.5-96.0)	60 (*Q1-Q3* 46.0-91.0)
Range	5-39	1-321	24-185
